# Dimethylfumarate Inhibits Colorectal Carcinoma Cell Proliferation: Evidence for Cell Cycle Arrest, Apoptosis and Autophagy

**DOI:** 10.3390/cells8111329

**Published:** 2019-10-28

**Authors:** Irina Kaluzki, Tsige Hailemariam-Jahn, Monika Doll, Roland Kaufmann, Panagiotis Balermpas, Nadja Zöller, Stefan Kippenberger, Markus Meissner

**Affiliations:** 1 Department of Dermatology, Venereology and Allergology, Goethe-University, 60438 Frankfurt am Main, Germany; 2 Department of Radiation Oncology, UniversitätsSpital, 8091 Zürich, Switzerland

**Keywords:** dimethylfumarate, colorectal carcinoma, p21, cell cycle arrest, cyclin D1, p62, LC3 I/II, caspase-8, autophagy

## Abstract

Recent studies have proven that Dimethylfumarate (DMF) has a marked anti-proliferative impact on diverse cancer entities e.g., on malignant melanoma. To explore its anti-tumorigenic potential, we examined the effects of DMF on human colon carcinoma cell lines and the underlying mechanisms of action. Human colon cancer cell line HT-29 and human colorectal carcinoma cell line T84 were treated with or without DMF. Effects of DMF on proliferation, cell cycle progression, and apoptosis were analyzed mainly by Bromodeoxyuridine (BrdU)- and Lactatdehydrogenase (LDH)-assays, caspase activation, flowcytometry, immunofluorescence, and immunoblotting. In addition, combinational treatments with radiation and chemotherapy were performed. DMF inhibits cell proliferation in both cell lines. It was shown that DMF induces a cell cycle arrest in G0/G1 phase, which is accompanied by upregulation of p21 and downregulation of cyclin D1 and Cyclin dependent kinase (CDK)4. Furthermore, upregulation of autophagy associated proteins suggests that autophagy is involved. In addition, the activation of apoptotic markers provides evidence that apoptosis is involved. Our results show that DMF supports the action of oxaliplatin in a synergetic manner and failed synergy with radiation. We demonstrated that DMF has distinct anti-tumorigenic, cell dependent effects on colon cancer cells by arresting cell cycle in G0/G1 phase as well as activating both the autophagic and apoptotic pathways and synergizes with chemotherapy.

## 1. Introduction

Colorectal cancer is the second most common malignant neoplasm in women and the third most common in men, thus forming the second most common cause of cancer death [1]. Despite relevant improvements in therapies, the prognosis of metastatic colorectal cancer patients remains poor. Treatment failure and serious side effects related to the available therapeutics demand therapies that increase survival rate with significantly less toxicity [2].

**Figure 1:**
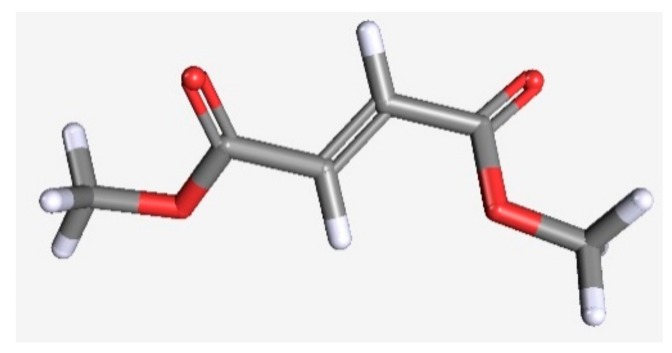
Chemical structure of Dimethylfumarate performed with Jmol (Jmol: an open-source Java viewer for chemical structures in 3D. http://www.jmol.org/).

Dimethylfumarate (DMF), a dimethyl ester of fumaric acid (Figure 1), has a low side effects profile and has been proven to be effective for the systemic treatment of inflammatory diseases such as psoriasis and multiple sclerosis.

After oral administration, DMF is quickly converted to its active metabolite monomethylfumarate (MMF) and the peak concentrations are detected 5–6 h later [3]. There is growing evidence that fumaric acid esters have a pronounced anti-tumorigenic potential in diverse cancer entities in both in vitro and in vivo trials. Recently, we reported on the inhibition of melanoma cell proliferation induced by DMF [4]. Furthermore, Kirlin et al. demonstrated that DMF inhibits colon cancer cell growth by inducing apoptosis [5]. This was complemented by Xie et al., who showed that DMF inhibits tumor proliferation via activation of the autophagic pathway and generation of reactive oxygen species [6]. Autophagy is a multi-step lysosomal degradation process and can result in autophagic cell death.

Autophagy in cancer and cancer treatment has been a controversial issue regarding its positive or negative role for tumor growth and seems dependent on the tumor entity as well as the compound inducing autophagy [7]. Additionally, Begleiter et al. revealed that a DMF diet prevents colon cancer in mice and rats by inducing phase II detoxifying enzymes [8]. Furthermore, it could be demonstrated that MMF is a metabolite of DMF enhanced CD56^+^ natural killer cell lysis of tumor cells by degranulation and upregulation of NKp46 and CD107a [9]. In addition, our group could demonstrate that DMF effectively inhibits angiogenesis and lymphangiogenesis in part by downregulation of a vascular endothelial growth factor receptor (VEGFR)-2 [10,11]. All of these results illustrate that DMF seems to have potential antitumorigenic effects. The underlying mechanisms of regulation are up to now not completely clear. Various authors demonstrated that DMF suppresses the activation as well as the nuclear translocation of Nuclear factor kappa B (NfkB) and therefore characterize p65 as a central target of DMF [12,13,14]. In addition, p21 and the cell cycle seem to be important targets of DMF [4,10,15].

There are now various clinical studies evaluating the effects of DMF and/or standard of care protocols in glioblastoma (NCT02337426 and NCT02490930) and refractory leukemia/lymphoma (NCT02784834). Thus, the first evidence that DMF induces apoptosis and autophagy in colorectal carcinoma cells has been provided. The underlying mechanisms of this action have yet to be determined. In this study, we investigated the in vitro influence of DMF on colon carcinoma cell proliferation and the underlying mechanisms of apoptosis, autophagy, and cell cycle regulation.

## 2. Material and Methods

### 2.1. Reagents

Dimethylfumarate, Triton X 100, chloroquine, propidium iodide and staurosporine were obtained from Sigma-Aldrich (Hamburg, Germany). Oxaliplatin was purchased from Abcam (Cambridge, UK) rapamycin from Merck’s Calbiochem (Darmstadt, Germany) and RNAse from Qiagen (Hilden, Germany).

### 2.2. Cell Culture

Human colorectal adenocarcinoma cell lines (HT-29 and T84 derived from a lung metastasis) were purchased from American type culture collection (ATCC) (Manassas, VA, USA). 

HT-29 cells were grown in McCoy’s 5A medium (Sigma-Aldrich, Hamburg, Germany) supplemented with 10% fetal calf serum (FCS, Greiner, Munich, Germany), 100 µg/mL streptomycin and 100 U/mL penicillin (Gibco/BRL, Karlsruhe, Germany). T84 cells were cultured in Dulbecco’s Modified Eagle Medium/Nutrient Mixture F-12 (DMEM/F-12, Gibco, Darmstadt, Germany) containing same supplements and 2 mM L-glutamine (Gibco/BRL, Karlsruhe, Germany), additionally. Both cell lines were maintained at 37 °C in a humidified atmosphere of 5% CO_2_ in air. The cell lines were grown to 60%–80% confluence before each passage using 0.25% trypsin/EDTA solution (Invitrogen, Karlsruhe, Germany).

Unless otherwise noted, in all experiments, cells were left to become adherent for 48 h after seeding and subsequently treated for 24 h with DMF or indicated substances before performing assay specific procedures and measurements.

### 2.3. Cell Proliferation and Cytotoxicity Assay

The effect of DMF on cell proliferation was measured by quantifying BrdU via a cell proliferation immunoassay from Roche Diagnostics (Grenzach, Germany). Forty-eight hours after seeding (1 × 10^4^ cells per 96-well), cells were incubated with BrdU and DMF at the indicated concentrations for 24 h. The cytotoxic potential of DMF was determined using an LDH-based cytotoxicity detection kit from Roche. Forty-eight hours after seeding (1 × 10^4^ cells per 96-well), the cells were incubated under serum-reduced conditions (1% FCS) with DMF for 24 h at the indicated concentrations.

### 2.4. Apoptosis Assays

The effect of DMF on apoptosis was analyzed using a Cell Death Detection ELISA PLUS-Kit and the Caspase 3/7 Assay from Roche Diagnostics (Grenzach, Germany). All assays were carried out according to the manufacturers’ instructions and 1 × 10^4^ cells were seeded per 96-well. 

### 2.5. Fluorescence-Activated Cell Sorting Analysis (FACS)

For cell cycle distribution analysis, cells (2.5 × 10^5^ cells) were incubated with 100 µM DMF and solvent as a control for 24 h after blocking the S phase of the cell-cycle by treatment with serum depleted medium for 24 h. The cells were fixed in ice-cold 70% ethanol. Cells were incubated in phosphate buffered saline (PBS) containing 40 µg/mL RNase A for 30 min at 37 °C and resuspended in PBS containing 50 µg/mL propidium iodide. Analysis of cell cycle was assessed by a BD FACScan Cytometer (Becton Dickinson, Franklin Lakes, NJ, USA).

### 2.6. Detection of Autophagy

The effect of DMF on autophagic activity was investigated using the CYTO-ID Autophagy Detection Kit from ENZO Life Sciences (Lörrach, Germany). Cells were collected (2.5 × 10^4^ per well) and stained according to the manufacturer’s manual. Cells treated with rapamycin 500 nM and chloroquine 10 µM were taken as positive control.

### 2.7. Combination of Oxaliplatin and Radiation

To analyze the anti-tumorigenic potential of the combination of DMF and oxaliplatin, as well as DMF and radiation concentrations of oxaliplatin and the Gy doses, were chosen cell lines specifically according to preliminary tests (Supplementary Figures S1–S4). In the combined treatment, oxaliplatin was added to the cells (0.5 × 10^4^ per well) 2 h after pre-incubation with BrdU and DMF or solvent, respectively, at indicated concentrations for another 22 h. To determine the additional impact of radiation, DMF pretreated cells (0.5 × 10^4^ per well) for 3 h were exposed to 0 Gray (Gy) vs. 2 Gy and incubated for additional 21 h for an overall treatment of 24 h. The anti-tumor effects were measured as inhibition of proliferation by quantifying BrdU as mentioned above. Irradiation with single doses of 2 Gy were carried out using a linear accelerator (SL-15, Elekta, Crawley, UK) with 6 MeV/100 cm focus-surface distance and a dose rate of 4 Gy/min. To ensure constant conditions, untreated cells were kept under equivalent surroundings in the irradiation control room.

### 2.8. Western Blot Analysis

Protein extracts were prepared as described previously [4]. Following Sodium dodecyl sulfate polyacrylamide gel electrophoresis (SDS-PAGE) and electroblotting, membranes were incubated with the following primary antibodies: anti-p21, anti-p27, anti-p16, CDK4, CDK6, cycline D1, cycline E, cycline B1, Light Chain3 (LC)3 AB I/II, Atg4B, p62, Atg3, Atg5, Atg7, Atg12, beclin-1, full length and cleaved caspases 3, 7, 8, 9, (phospho-)p38, (phospho-)p44/42, (phospho-)rb, (phospho-)akt, and anti-tubulin (Cell Signaling, Danvers, MA, USA). Primary antibody application was followed by incubation with horseradish peroxidase-conjugated secondary antibodies (anti-mouse and anti-rabbit IgG, Amersham, Uppsala, Sweden; anti-goat, Dako, Glostrup, Denmark). Blots were developed using an enhanced chemiluminescence detection system (ECL) (Amersham) according to the manufacturer’s instructions.

### 2.9. Statistical Analysis

The data are expressed as the mean ± standard error of the mean (SEM) from at least three independent experiments. Statistical analyses were performed using the one-Way ANOVA test. As a statistical program, we used QtiPlot 0.9.9. * *p* < 0.05.

## 3. Results

### 3.1. DMF Has No Cytotoxic Effects and Inhibits Colon Carcinoma Cell Proliferation

**Figure 2:**
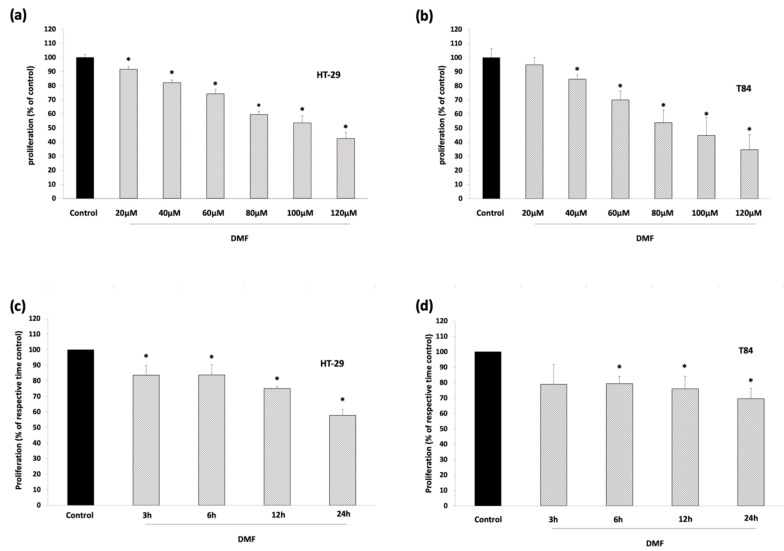

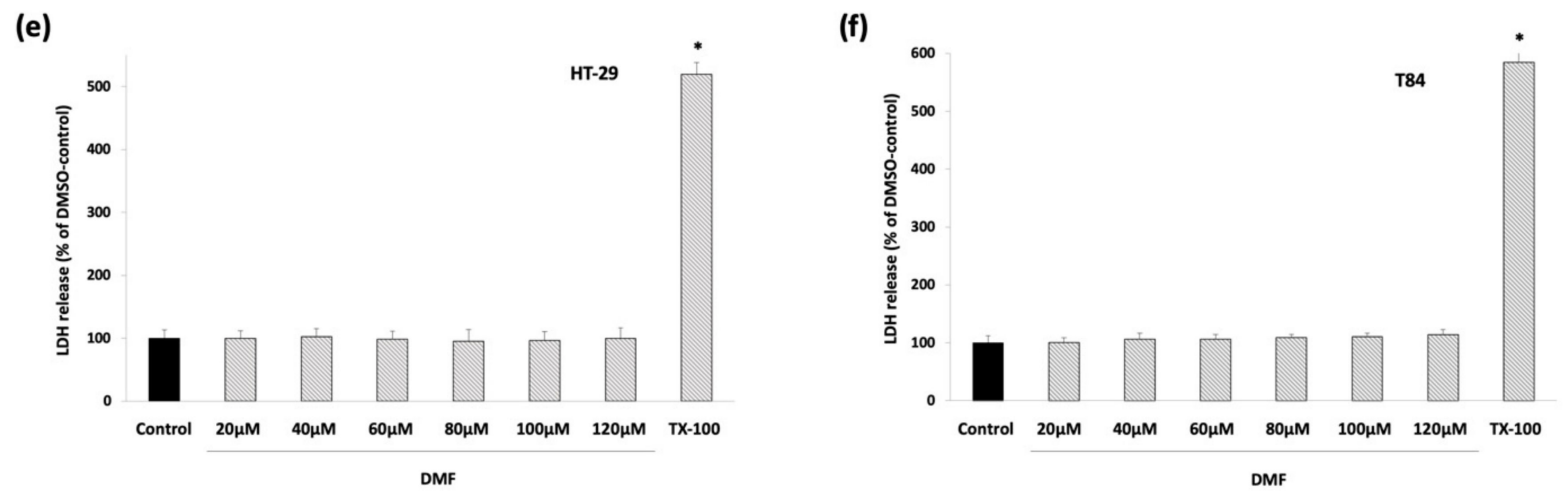
DMF suppresses colon carcinoma cell proliferation but does not display cytotoxic effects. (**a**,**b**) proliferation assay: HT-29 and T84 were treated for 24 h with the indicated concentrations of DMF. Dimethylsulfoxide (DMSO) 0.2% solvent served as control; (**c**,**d**) proliferation assay: HT-29 and T84 were treated with 100 µM DMF for the indicated time. DMSO 0.2% solvent served as control; (**e**,**f**) cytotoxicity-assay: HT-29 and T84 were treated for 24 h with the indicated concentrations of DMF. DMSO 0.2% solvent served as negative control, Triton X as a positive control. Mean values from at least three independent experiments are shown as mean ± SD. * *p* < 0.05: significant.

We examined the effect of DMF on cell proliferation and investigated its cytotoxicity using two colorectal adenocarcinoma cell lines, HT-29 and T84, revealing that it inhibited colorectal carcinoma (CRC) proliferation in a concentration- and time-dependent manner, as determined by BrdU assay (Figure 2a–d)

DMF significantly reduced proliferation up to 57% in HT-29 and up to 65% in T84 cells. In a time-modified set-up, 100 µM DMF showed an inhibition of cell proliferation by 16% in HT-29 and 21% in T84 after only 3 h of treatment, followed by gradually progressive inhibition to 42% and 30%, respectively, after 24 h.

These results were not conveyed through cytotoxic effects because DMF did not significantly increase LDH (Figure 2e,f).

### 3.2. DMF Induces G0/G1 Cell Cycle Arrest in HT-29 and Augments Sub-G0/G1 Phase in T84

**Figure 3:**
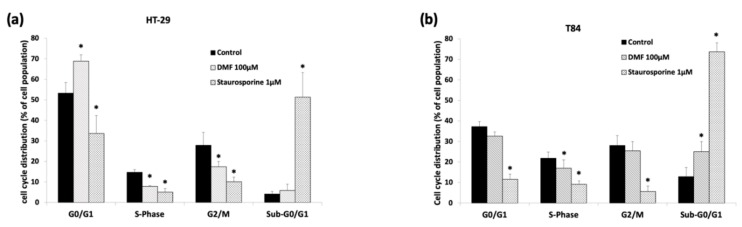
DMF induces G0/G1 arrest in HT-29 and increases the sub-G0/G1 phase in T84. Analysis of cell cycle distribution by FACS using propidium iodide-stained colon carcinoma cell lines (**a**) HT-29 treated with 100 µM DMF for 24 h; (**b**) T84 were treated with 100 µM DMF for 24 h. Positive control: Staurosporine 1 µM; Negative control: DMSO 0.2%. Data displayed are the mean values of at least three independent experiments and results are shown as mean ± SD. * *p* < 0.05: significant.

Using the FACS analysis with propidium iodide-stained HT-29 and T84 cells, we found that DMF treatment significantly increased the G0/G1 phase distribution from 53% to 69% in HT-29 with subsequent reduction of cells in S and G2/M phase, demonstrating G0/G1 cell cycle arrest (Figure 3a). 

Surprisingly, this effect could not be seen in T84 cells despite similarly decreased proliferation under DMF treatment. Instead, the FACS analysis revealed an augmentation of DMF treated T84 cells in the sub-G0/G1 phase from 13% to 25%, indicating that cell death mechanisms were involved in the anti-tumorigenic action of DMF (Figure 3b). 

**Figure 4:**
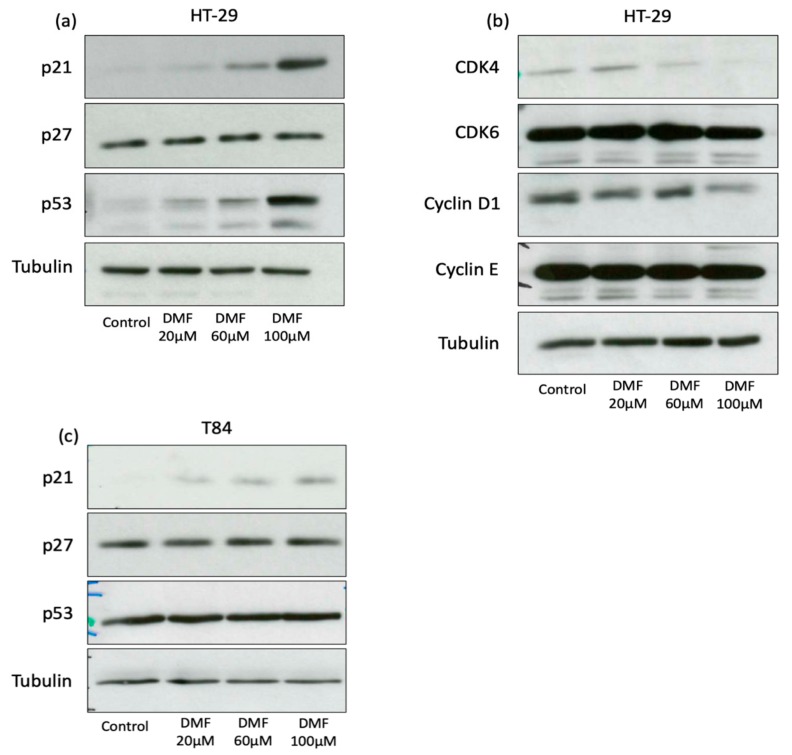
DMF induces p21 in both cell lines and p53 in HT-29 and suppresses CDK4 and cyclin D1 protein expression only in HT-29 cells. Representative Western blot analyses of (**a**,**b**) HT-29 treated for 24 h with DMF in the indicated concentrations and (**c**) T84 cells treated for 24 h with DMF in the indicated concentrations. DMSO 0.2% served as a solvent control. Comparable results were obtained from at least three independent experiments.

These data show that the specific cell cycle arrest phase was cell line dependent. To determine the underlying mechanisms of cell cycle arrest, we examined the expression of important cell cycle regulators. P21 levels increased in a concentration-dependent manner in HT-29 cells, whereas p27 levels were not changed (Figure 4a). The inhibitory function of p21 to cell cycle could be further illustrated. The increase in p21 expression was accompanied by p53 protein induction. The expression of cyclin D1, an important driver of the G1/S phase transition and CDK4, one of its complex partners, was suppressed in a dose-dependent manner (Figure 4b).

Expression of the cell cycle regulators p27, cyclin E and CDK6 did not change after DMF treatment in HT-29. In T84, an increase in p21 expression under DMF could be demonstrated, whereas p27 and p53 were not influenced by DMF (Figure 4c).

### 3.3. DMF Induces Cell Death-Related Mechanisms in T84

**Figure 5:**
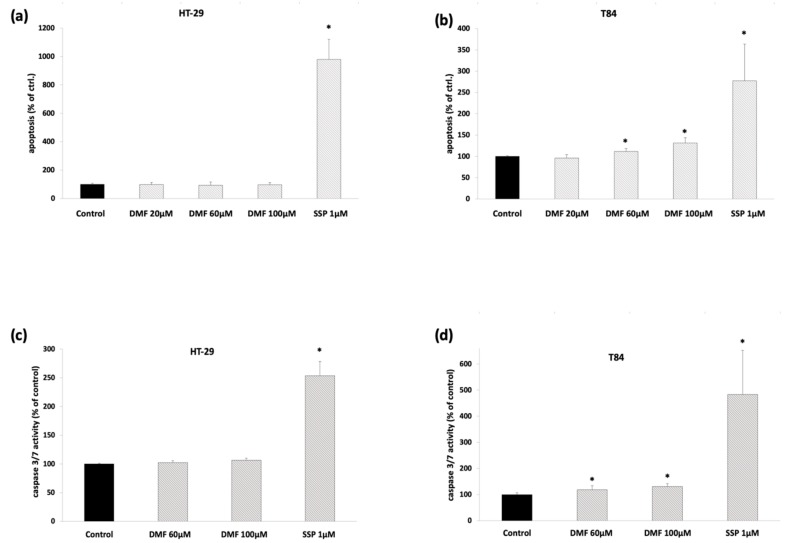
DMF induces apoptosis and caspase-3/7 activation in T84 but not HT-29 cells. Analysis of apoptosis using the Cell Death Detection Kit. (**a**) HT-29 cells were treated for 24 h with DMF at the indicated concentrations; (**b**) T84 cells were treated for 24 h with DMF at the indicated concentrations. Analysis of caspase-3/7 activity; (**c**) HT-29 cells were treated for 24 h with DMF at the indicated concentrations; and (**d**) T84 cells were treated for 24 h with DMF at the indicated concentrations. Mean values from at least three independent experiments are shown as mean ± SD. * *p* < 0.05: significant. As a positive control, staurosporine (SSP) 1 µM was used. DMSO 0.2% served as a solvent control.

To rule out cell death-related mechanisms as pointed out by the cell cycle analysis in T84, we investigated the DMF-induced release of cytoplasmic histone-associated DNA fragments and caspase-3/7 activity. We found that DMF modestly but significantly induced apoptotic cell death and increased caspase-3/7 activity in a concentration-dependent manner in T84 (Figure 5a–d), but not in HT-29 cells. 

### 3.4. DMF Induces Autophagy and Decreases Activity of Cellular Senescence

**Figure 6:**
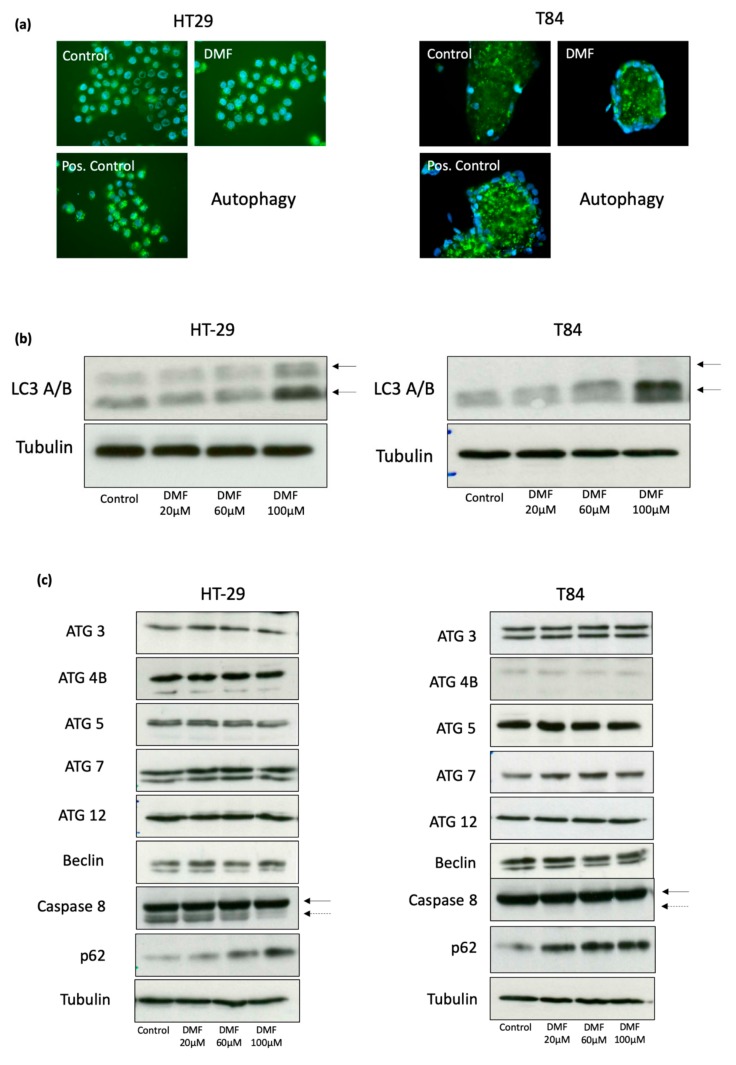
DMF induces autophagy in HT-29 and T84 colon carcinoma cell lines. (**a**) representative immunofluorosecence of autophagy using Cyto-ID^®^ assay. Cells were treated with DMF (100 µM) or solvent for 24 h. As a positive control Rapamycin (500 nM) + Chloroquine (10 µM) were used; (**b**) representative Western blot analyzes of LC3 A/B. Cells were treated for 24 h with vehicle or DMF for the indicated concentrations. The two arrows mark the type I (upper arrow) and type II (lower arrow) LC3A/B; (**c**) representative Western blot analysis from different autophagy associated proteins. Cells were treated for 24 h with vehicle or DMF for the indicated concentrations. Comparable results were obtained from at least three independent experiments.

To reveal further potential mechanisms of proliferation inhibition induced by DMF, we next explored autophagic and senescent activities. Visualization of the autophagic vacuoles was performed by immunofluorescence. We found that DMF increased autophagic activity in both HT-29 and T84 cells (Figure 6a). 

These results were confirmed by significantly elevated protein levels of LC3 A/B, a major constituent of the autophagosome in immunofluoroscence and Western blot (Figure 6a,b). Furthermore, we demonstrated an increase of p62 (SQSTM1) in both cell lines, which functions as an autophagosome cargo protein via binding to LC3 B and target proteins for degradation (Figure 6c). 

In addition, we could show a suppression of the activated form of caspase-8 in HT-29 cells, whereas, in T84, caspase-8 stayed unchanged. Other autophagy-related proteins Atg3, Atg4B, Atg5, Atg7, Atg12, and beclin-1 remained unchanged in our setting. 

### 3.5. DMF Shows Synergetic Effect in Combination with Oxaliplatin but Does Not Potentiate Radiation Response

**Figure 7:**
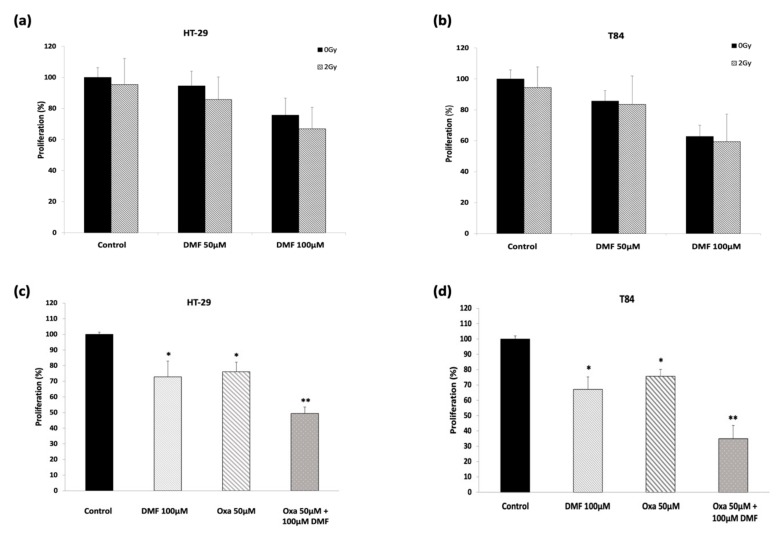
Combinational treatment with DMF and radiation or oxaliplatin. Proliferation assay for the DMF/radiation treatment (**a**) HT-29 and (**b**) T84 cell lines. DMF pretreated cells (3 h) were exposed to 0 Gy vs. 2 Gy and incubated for additional 21 h for an overall treatment timed of 24 h. Proliferation assay for DMF/oxaliplatin treatment in (**c**) HT-29 and (**d**) T84 cell lines. Oxaliplatin was added 2 h after pre-incubation with DMF or solvent, respectively, at indicated concentrations for another 22 h. Mean values from at least three independent experiments are shown as mean ± SD. *p* < 0.05: significant. * significant compared to untreated control. ** significant compared to single treatments and untreated control.

To determine whether radiation ameliorates DMF-induced anti-tumor effects, simultaneous treatment with radiation and DMF was performed. Additional impact of radiation on colon carcinoma proliferation was only slight and not significant in both cell lines (Figure 7a,b).

Furthermore, we explored the anti-proliferative effect of DMF in combination with oxaliplatin, which is used as an antineoplastic drug in colorectal cancer chemotherapy (Figure 7c,d). Our results show that DMF significantly fortifies the action of oxaliplatin in a synergetic manner. While DMF and oxaliplatin reduced proliferation by approximately a quarter, the combinational treatment reduced proliferation up to two-thirds.

## 4. Discussion

Different authors have provided the first evidence that DMF treatment in vitro leads to growth inhibition of colon cancer of human and animal origin [5,6,16]. Furthermore, in vivo experiments with mouse models also suggest a colon cancer protective effect [8,17]. The underlying mechanisms are only partly clarified.

In this study, we revealed that DMF inhibited the proliferation of two human colon carcinoma cell lines via a cell line-dependent induction of G0/G1 cell cycle arrest, apoptosis, and autophagy in a concentration- and time-dependent manner. We also showed increased expression of the tumor suppressor p21 in both cell lines and suppressed expression of the cell cycle inductor cyclin D1 and its complex partner CDK4 in HT-29 cells as the likely underlying mechanism of G0/G1 arrest. In addition, we demonstrated that DMF induced apoptosis in T84 cells. Furthermore, we showed a DMF-induced autophagic activity in both HT-29 and T84 cells. This was substantiated by elevated protein levels of LC3 A/B and p62 in both cell lines and reduced caspase-8 activity in HT-29. Moreover, we could demonstrate that the combinational treatment with DMF and chemotherapy acts synergistic, whereas the combination of radiotherapy and DMF has no synergistic effects.

Recently, we reported that DMF inhibits the proliferation of melanoma cell lines via a concentration-dependent induction of G0/G1 and G2/M arrest in a p21-dependent manner [4]. Our data bolster this finding in colon carcinoma cells, since we revealed a concentration-dependent increase in p21 expressions in both cell lines. Furthermore, we observed a G0/G1 cell cycle arrest in HT-29 cells, which is in line with the results in vascular smooth muscle cells, pre-adipocytes and lymphendothelial cells where DMF was shown to induce G0/G1 arrest via induction of p21 [4,10,15,18]. Thus, the induction of a p21 dependent G0/G1 arrest seems to be an important way of DMF dependent regulation in various cell and tumor entities. In colon carcinoma, the induction of p21 is an important way of cell cycle arrest. Singh et al. demonstrated that the heparanase inhibitor PG545 effectively inhibits colon carcinoma cell proliferation via p21 induction and G0/G1 arrest [19]. Afrin et al. showed in HCT-116 cells that manuka honey, which seems to have a cancer preventive effect, induces p21 and G2/M cell cycle arrest [20]. 

Besides the induction of p21, we could demonstrate a suppressed expression of the cell cycle regulators cyclin D1 and its complex partner CDK4 in HT-29 cells. The downregulation of cyclin D1 and CDK4 is an important and effective way of cell cycle arrest. Singh et al. demonstrated that resveratrol inhibits cyclin D1 and CDK4 expression in human colon carcinoma cells inducing G0/G1 arrest [21]. Rezaei et al. showed comparable results in breast cancer cells for pericarp extract [22]. In addition, in HT-29 cells, we could demonstrate the induction of p53. P53 is an important regulator of the cell cycle and is known to regulate p21. Hence, the p53 induction in HT-29 is in line with the p21 induction and cell cycle arrest. In T84 cells, p53 was not induced by DMF and therefore p21 might be regulated p53 independently. Comparable results could be demonstrated by us in melanoma cells, where the knockdown of p53 did not influence the G0/G1 cell cycle arrest [4].

**Figure 8:**
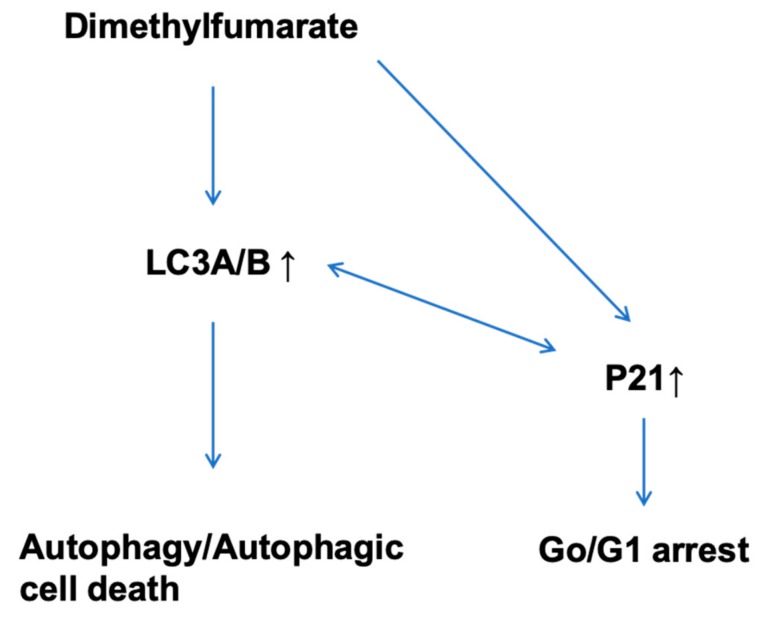
Possible DMF dependent way of regulation in colon carcinoma cell lines.

In T84 cells, blocked cell cycle boundaries could not be demonstrated. Instead, DMF treatment almost doubled the sub-G0/G1 phase distribution from 13% to 25%, indicating apoptotic mechanisms. This result was corroborated by further apoptosis-related findings, such as DMF-induced increase of apoptotic nucleosomes and caspase-3/7 activity in T84. The induction of apoptosis by DMF has been demonstrated in several cancer entities before, as for example in melanoma cells [4,23], and hematopoetic tumor cells [24]. The differences in the underlying mechanisms of proliferation suppression in HT-29 and T84 might, in addition, depend on the fact that HT-29 is a primary site tumor and T84 is derived from a metastasis and therefore are differently affected by DMF. In prospective analysis, a set of primary and metastatic tumors should be evaluated concerning the differences of regulation by DMF treatment. Contrary to our results, Kirlin et al. reported in HT-29 cells DMF-induced apoptosis, which was measured by loss of adherence, nuclear fragmentation, and increased caspase-3-like activity in response to DMF treatment [6]. We could not reproduce these results. In our experiments with three different ways of apoptosis analysis, neither the release of cytoplasmic histone associated DNA-fragments nor a DMF induced caspase-3/7 activity nor a subG0 phase increase in the cell cycle analysis could be demonstrated. Kirlin et al. combined in part DMF treatment with the differentiation agent sodium butyrate, which might explain the different results. In addition, the investigators themselves remarked a relatively slow and probably incomplete DMF-prompted development of apoptosis even at 24 h. Different culture conditions might also add to different effects. Moreover, Xie et al. did not exclude the possibility of DMF-induced cell apoptosis in HCT116 (human) and CT26 (murine) colon cancer cells, but found DMF not to significantly affect apoptotic parameters, such as the expression of bcl-2, bax, and cleaved caspase-3 protein [6]. In addition, Xie et al. demonstrated that DMF is able to increase autophagy in colon cancer cells. This specific effect could be reversed in the murine CT26 cells by treatment with the inhibitor of autophagy DMH1, but, interestingly, it did not affect the DMF-induced decrease in cell viability, which might indicate that autophagy is not the exclusive way of regulation. We received an analogue outcome of enhanced autophagic activity under DMF treatment in both HT-29 and T84 cell lines detecting an increased amount of autophagic vacuoles and LC3A/B by immunofluorescence analysis. These findings were accompanied by significantly elevated protein levels of LC3 A/B and p62 and reduced expression of activated caspase-8. The role of autophagy in cancer and cancer treatment has been a controversial issue regarding its positive or negative role for tumor growth. It has been recognized that the effect depends on the tissue, developmental stage of the tumor, and degree of autophagic activity. Ma et al. could illustrate the double-edged effects of Tanshinone-induced autophagy on osteosarcoma cells [25]. With low dosing and shorter treatment periods autophagic signaling activated damage repair mechanisms, whereas, on high-dose treatment over longer periods, autophagy contributed to apoptosis. Recently, Chen et al. showed that Apigenin effectively inhibits tumorigenesis in vivo and in vitro by the combination of apoptosis and autophagy in colon carcinoma [26]. Comparable results were demonstrated by Son et al. or Gao et al. in colon carcinoma cell lines [27,28]. Interestingly, Li et al. demonstrated in prostate cancer cells that the induction of autophagy induces p21 [29]. Comparable results were demonstrated by Wang et al. in pancreatic cancer cells [30]. Fan et al. as well as Di Giacomo et al. contrarily demonstrated that the induction of p21 induces autophagy [31,32]. Thus, it seems there is a close regulatory relation between p21 and autophagy which might be cell entity specific. The possible DMF dependent regulatory pathway is depicted in Figure 8. 

These findings suggest that the anti-tumor effects of DMF in T84 cells are induced by apoptosis as well as autophagy. Hanot et al. and Boivin et al. demonstrated that the treatment of head and neck cancer cells with DMF sensitizes them to radiation and induces cell death [33,34]. Comparable results were shown by Booth et al. in glioblastoma [35]. To prove whether this effect can be transferred to colon carcinoma, we performed combinational treatments with DMF and radiation for 24 h. Here, we could demonstrate no synergistic effects. Of course, on the one hand, it might be possible that longer post radiation times demonstrate further effects, but, on the other hand, the effects of DMF on proliferation appear as early as after 3 h. Future in vivo experiments might include longer treatment times. In contrast, the combination of chemotherapy (oxaliplatin) and DMF displayed a profound and significant synergism concerning proliferation inhibition. In future experiments, it might be interesting to analyze whether the synergism is based more on cytotoxicity, apoptosis, or cell cycle inhibition. Booth et al. could demonstrate comparable results in freshly isolated glioblastoma patient cells where the combination of proteasome inhibitors and DMF significantly increased tumor cell death, in part by inducing autophagy [35]. There are now various clinical studies evaluating the effects of DMF and/or standard of care protocols in glioblastoma (NCT02337426 and NCT02490930) and refractory leukemia/lymphoma (NCT02784834).

## 5. Conclusions

In summary, we have elucidated the underlying mechanisms of DMF-induced apoptosis, autophagy, and cell cycle inhibition in colon carcinoma cell lines. We demonstrated in particular the activation of caspase-8 and upregulation of p21, LC3 A/B and p62. In addition, in HT-29 cells, we could show the suppression of cyclin D1 and CDK4, which, in part, explains the G0/G1 cell cycle arrest. These data now explain, in part, the anti-tumorigenic action of DMF in colorectal cancer and might be the foundation for further in vivo analysis.

## Supplementary Materials

The following are available online at https://www.mdpi.com/2073-4409/8/11/1329/s1, Figure S1. Influence of different irradiation doses on the proliferation of HT-29 cells. Figure S2. Influence of different irradiation doses on the proliferation of T84 cells. Figure S3. Influence of different Oxaliplatin concentrations on the proliferation of HT-29 cells. Figure S4. Influence of different Oxaliplatin concentrations on the proliferation of T84 cells.

## Abbreviations

DMF	Dimethylfumarate
BrdU	Bromodeoxyuridine
LDH	lactatedehydrogenase
DMSO	Dimethylsulfoxide
FCS	fetal calf serum
NAC	*N*-acetylcystein
CRC	colorectal carcinoma
ROS	reactive oxygen species

## References

[1] Bray F., Ferlay J., Soerjomataram I., Siegel R.L., Torre L.A., Jemal A. (2018). Global cancer statistics 2018: GLOBOCAN estimates of incidence and mortality worldwide for 36 cancers in 185 countries. CA A Cancer J. Clin..

[2] Van Cutsem E., Cervantes A., Adam R., Sobrero A., Van Krieken J.H., Aderka D., Aranda Aguilar E., Bardelli A., Benson A., Bodoky G. (2016). ESMO consensus guidelines for the management of patients with metastatic colorectal cancer. Ann. Oncol..

[3] Werdenberg D., Joshi R., Wolffram S., Merkle H.P., Langguth P. (2003). Presystemic metabolism and intestinal absorption of antipsoriatic fumaric acid esters. Biopharm. Drug Dispos..

[4] Kaluzki I., Hrgovic I., Hailemariam-Jahn T., Doll M., Kleemann J., Valesky E.M., Kippenberger S., Kaufmann R., Zoeller N., Meissner M. (2016). Dimethylfumarate inhibits melanoma cell proliferation via p21 and p53 induction and bcl-2 and cyclin B1 downregulation. Tumor Boil..

[5] Kirlin W.G., Cai J., Delong M.J., Patten E.J., Jones D.P. (1999). Dietary Compounds That Induce Cancer Preventive Phase 2 Enzymes Activate Apoptosis at Comparable Doses in HT29 Colon Carcinoma Cells. J. Nutr..

[6] Xie X., Zhao Y., Ma C.-Y., Xu X.-M., Zhang Y.-Q., Wang C.-G., Jin J., Shen X., Gao J.-L., Li N. (2015). Dimethyl fumarate induces necroptosis in colon cancer cells through GSH depletion/ROS increase/MAPKs activation pathway. Br. J. Pharmacol..

[7] Mizushima N. (2018). A brief history of autophagy from cell biology to physiology and disease. Nature.

[8] Begleiter A., Sivananthan K., Curphey T.J., Bird R.P. (2003). Induction of NAD(P)H quinone: oxidoreductase1 inhibits carcinogen-induced aberrant crypt foci in colons of Sprague-Dawley rats. Cancer Epidemiol. Biomark. Prev..

[9] Vego H., Sand K.L., Høglund R.A., Fallang L.E., Gundersen G., Holmøy T., Maghazachi A.A. (2016). Monomethyl fumarate augments NK cell lysis of tumor cells through degranulation and the upregulation of NKp46 and CD107a. Cell Mol. Immunol..

[10] Valesky E.M., Hrgovic I., Doll M., Wang X., Pintér A., Kleemann J., Kaufmann R., Kippenberger S., Meissner M. (2016). Dimethylfumarate effectively inhibits lymphangiogenesis via p21 induction and G1 cell cycle arrest. Exp. Dermatol..

[11] Meissner M., Doll M., Hrgovic I., Reichenbach G., König V., Hailemariam-Jahn T., Gille J., Kaufmann R. (2011). Suppression of VEGFR2 expression in human endothelial cells by dimethylfumarate treatment: Evidence for anti-angiogenic action. J. Invest. Dermatol..

[12] Loewe R., Holnthoner W., Gröger M., Pillinger M., Gruber F., Mechtcheriakova D., Hofer E., Wolff K., Petzelbauer P. (2002). Dimethylfumarate inhibits TNF-induced nuclear entry of NF-kappa B/p65 in human endothelial cells. J. Immunol..

[13] Gerhardt S., König V., Doll M., Hailemariam-Jahn T., Hrgovic I., Zöller N., Kaufmann R., Kippenberger S., Meissner M. (2015). Dimethylfumarate protects against TNF-α-induced secretion of inflammatory cytokines in human endothelial cells. J. Inflamm. (Lond.).

[14] Yamazoe Y., Tsubaki M., Matsuoka H., Satou T., Itoh T., Kusunoki T., Kidera Y., Tanimori Y., Shoji K., Nakamura H. (2009). Dimethylfumarate inhibits tumor cell invasion and metastasis by suppressing the expression and activities of matrix metalloproteinases in melanoma cells. Cell Biol. Int..

[15] Oh C.J., Park S., Kim J.-Y., Kim H.-J., Jeoung N.H., Choi Y.-K., Go Y., Park K.-G., Lee I.-K. (2014). Dimethylfumarate attenuates restenosis after acute vascular injury by cell-specific and Nrf2-dependent mechanisms. Redox Boil..

[16] Odom R.Y., Dansby M.Y., Rollins-Hairston A.M., Jackson K.M., Kirlin W.G. (2009). Phytochemical induction of cell cycle arrest by glutathione oxidation and reversal by N-acetylcysteine in human colon carcinomacarcinoma cells. Nutr. Cancer.

[17] Ma Z.-G., Ma R., Xiao X.-L., Zhang Y.-H., Zhang X.-Z., Hu N., Gao J.-L., Zheng Y.-F., Dong D.-L., Sun Z.-J. (2016). Azo polymeric micelles designed for colon-targeted dimethyl fumarate delivery for colon cancer therapy. Acta Biomater..

[18] Kang H.-J., Seo H.-A., Go Y., Oh C.J., Jeoung N.H., Park K.-G., Lee I.-K. (2013). Dimethylfumarate Suppresses Adipogenic Differentiation in 3T3-L1 Preadipocytes through Inhibition of STAT3 Activity. PLoS ONE.

[19] Singh P., Blatt A., Feld S., Zohar Y., Saadi E., Barki-Harrington L., Hammond E., Ilan N., Vlodavsky I., Chowers Y. (2017). The Heparanase Inhibitor PG545 Attenuates Colon Cancer Initiation and Growth, Associating with Increased p21 Expression. Neoplasia.

[20] Afrin S., Giampieri F., Gasparrini M., Forbes-Hernández T.Y., Cianciosi D., Rodríguez P.R., Amici A., Quiles J.L., Battino M. (2018). The inhibitory effect of Manuka honey on human colon cancer HCT-116 and LoVo cell growth. Part 1: the suppression of cell proliferation, promotion of apoptosis and arrest of the cell cycle. Food Funct..

[21] Singh S.K., Banerjee S., Acosta E.P., Lillard J.W., Singh R. (2017). Resveratrol induces cell cycle arrest and apoptosis with docetaxel in prostate cancer cells via a p53/ p21WAF1/CIP1 and p27KIP1 pathway. Oncotarget.

[22] Rezaei P.F., Fouladdel S., Ghaffari S.M., Amin G., Azizi E. (2012). Induction of G1 cell cycle arrest and cyclin D1 downregulation in response to pericarp extract of Baneh in human breast cancer T47D cells. DARU J. Pharm. Sci..

[23] Loewe R., Valero T., Kremling S., Pratscher B., Kunstfeld R., Pehamberger H., Petzelbauer P. (2006). Dimethylfumarate Impairs Melanoma Growth and Metastasis. Cancer Res..

[24] Tsubaki M., Ogawa N., Takeda T., Sakamoto K., Shimaoka H., Fujita A., Itoh T., Imano M., Satou T., Nishida S. (2014). Dimethyl fumarate induces apoptosis of hematopoietic tumor cells via inhibition of NF-κB nuclear translocation and downregulation of Bcl-xL and XIAP. Biomed. Pharmacother..

[25] Ma K., Zhang C., Huang M.-Y., Guo Y.-X., Hu G.-Q. (2016). Crosstalk between Beclin-1-dependent autophagy and caspase-dependent apoptosis induced by tanshinone IIA in human osteosarcoma MG-63 cells. Oncol. Rep..

[26] Chen X., Xu H., Yu X., Wang X., Zhu X., Xu X. (2019). Apigenin inhibits in vitro and in vivo tumorigenesis in cisplatin-resistant colon cancer cells by inducing autophagy, programmed cell death and targeting m-TOR/PI3K/Akt signalling pathway. J. BUON.

[27] Son Y., An Y., Jung J., Shin S., Park I., Gwak J., Ju B.G., Chung Y., Na M., Oh S. (2019). Protopine isolated from Nandina domestica induces apoptosis and autophagy in colon cancer cells by stabilizing p53. Phytother. Res..

[28] Gao G.-Y., Ma J., Lu P., Jiang X., Chang C. (2018). Ophiopogonin B induces the autophagy and apoptosis of colon cancer cells by activating JNK/c-Jun signaling pathway. Biomed. Pharmacother..

[29] Li X., Li X., Wang J., Ye Z., Li J.C. (2012). Oridonin upregulates expression of P21 and induces autophagy and apoptosis in human prostate cancer cells. Int. J. Biol Sci..

[30] Wang Y., Kuramitsu Y., Baron B., Kitagawa T., Tokuda K., Akada J., Nakamura K. (2015). CGK733-induced LC3 II formation is positively associated with the expression of cyclin-dependent kinase inhibitor p21Waf1/Cip1 through modulation of the AMPK and PERK/CHOP signaling pathways. Oncotarget.

[31] Fan J.D., Lei P.J., Zheng J.Y., Wang X., Li S., Liu H., He Y.L., Wang Z.N., Wei G., Zhang X. (2015). The selective activation of p53 target genes regulated by SMYD2 in BIX-01294 induced autophagy-related cell death. PLoS ONE.

[32] Di Giacomo V., Di Valerio V., Rapino M., Bosco D., Cacciatore I., Ciulla M., Marrazzo A., Fiorito J., Di Stefano A., Cataldi A. (2015). MRJF4, a novel histone deacetylase inhibitor, induces p21 mediated autophagy in PC3 prostate cancer cells. Cell Mol. Biol..

[33] Hanot M., Boivin A., Malésys C., Beuve M., Colliaux A., Foray N., Douki T., Ardail D., Rodriguez-Lafrasse C. (2012). Glutathione Depletion and Carbon Ion Radiation Potentiate Clustered DNA Lesions, Cell Death and Prevent Chromosomal Changes in Cancer Cells Progeny. PLoS ONE.

[34] Boivin A., Hanot M., Malésys C., Maalouf M., Rousson R., Rodriguez-Lafrasse C., Ardail D. (2011). Transient Alteration of Cellular Redox Buffering before Irradiation Triggers Apoptosis in Head and Neck Carcinoma Stem and Non-Stem Cells. PLoS ONE.

[35] Booth L., Cruickshanks N., Tavallai S., Roberts J.L., Peery M., Poklepovic A., Dent P. (2014). Regulation of dimethyl-fumarate toxicity by proteasome inhibitors. Cancer Boil. Ther..

