# Supplementary material for: Dimethylfumarate Inhibits Colorectal Carcinoma Cell Proliferation: Evidence for Cell Cycle Arrest, Apoptosis and Autophagy
Source: Cells. 2019 Oct 28;8(11):1329. doi: 10.3390/cells8111329 (PMC6912700; doi:10.3390/cells8111329)
Supplement: Supplementary file 1 [file cells-08-01329-s001.pdf]

Supplementary Figures

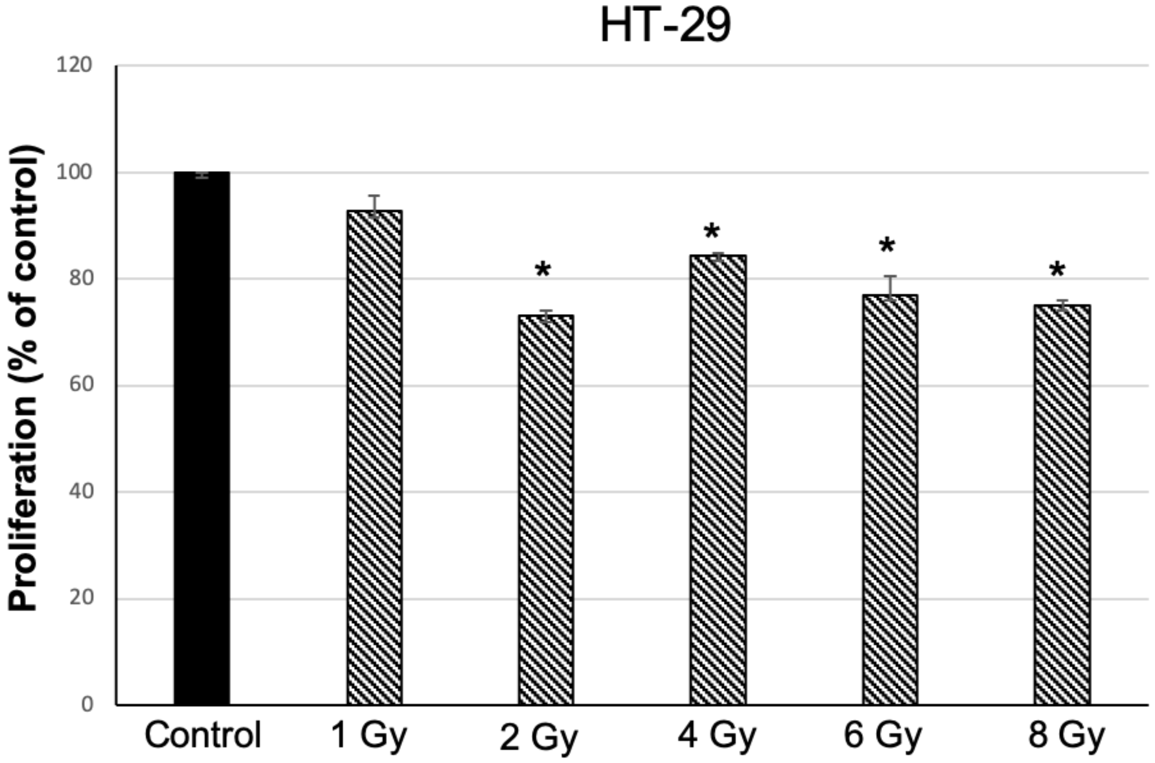

Figure 1. Influence of different irradiation doses on the proliferation of HT-29 cells. Cells were exposed to the indicated Gy doses and grown for 24 h. Mean values from at least three independent experiments are shown as mean ± SD. $*p < 0.05$.

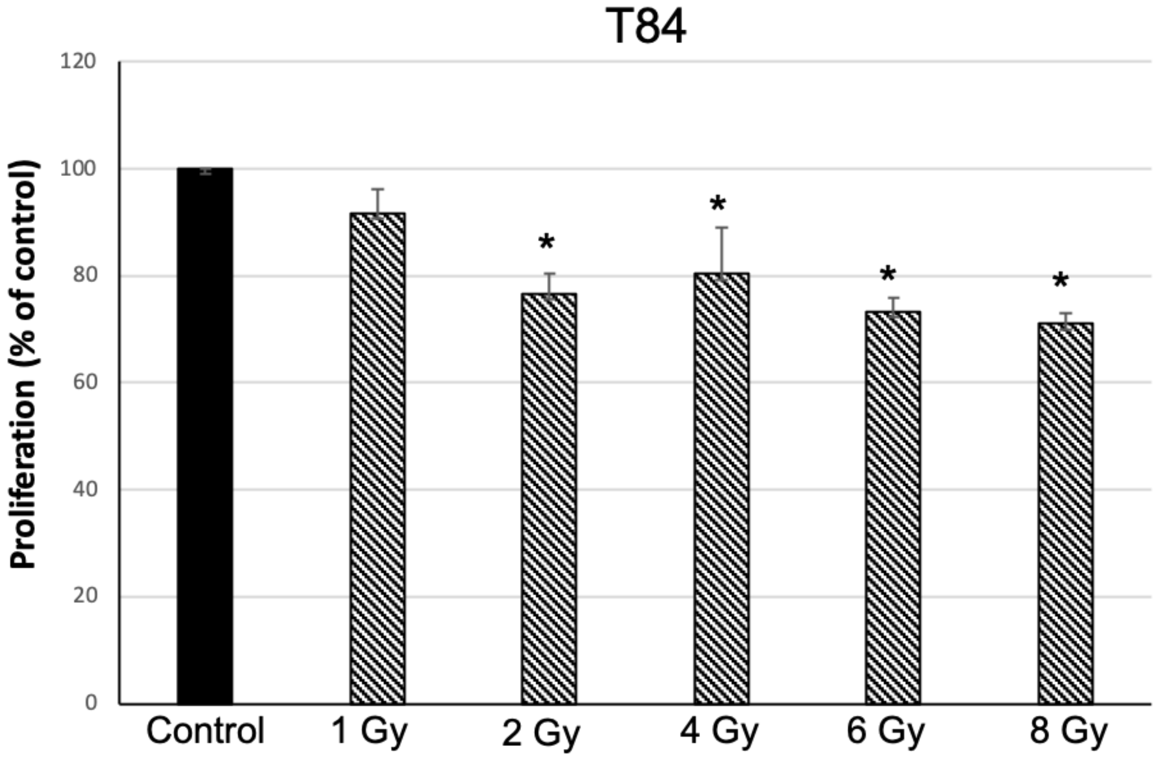

Figure 2. Influence of different irradiation doses on the proliferation of T84 cells. Cells were exposed to the indicated Gy doses and grown for 24 h. Mean values from at least three independent experiments are shown as mean ± SD. $*p < 0.05$.

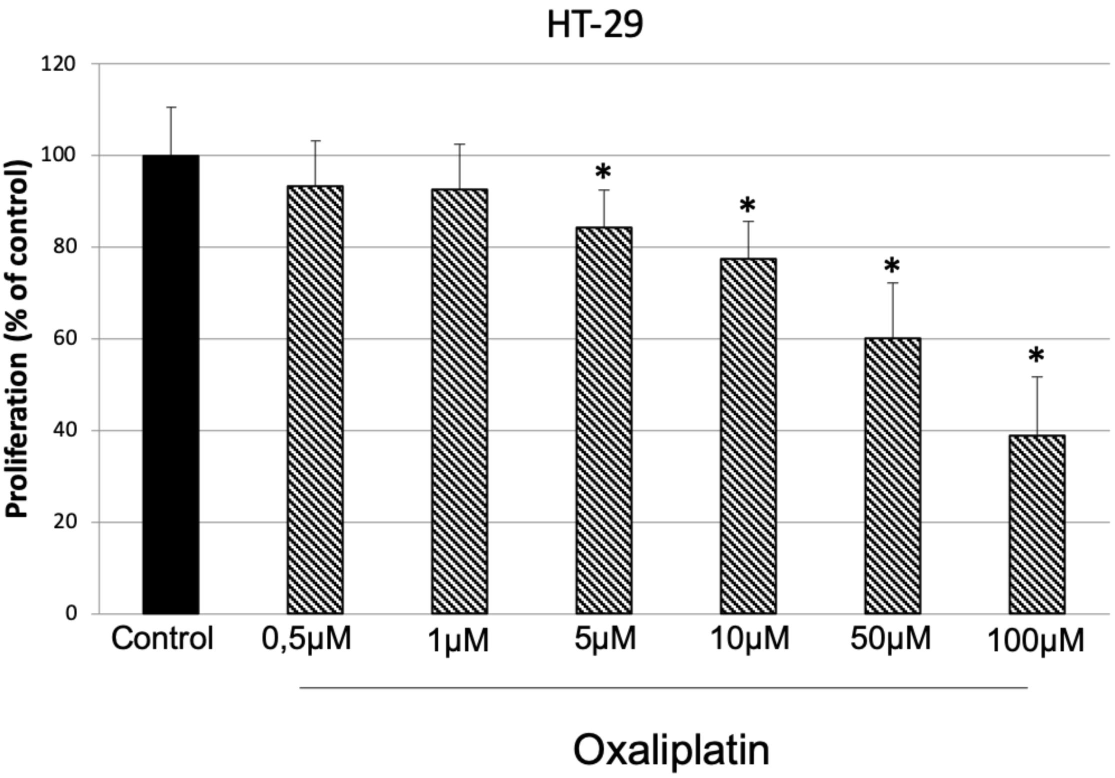

Figure 3. Influence of different Oxaliplatin concentrations on the proliferation of HT-29 cells. Oxaliplatin was added at the indicated concentrations for another 24 h. Mean values from at least three independent experiments are shown as mean ± SD. *$p < 0.05$.

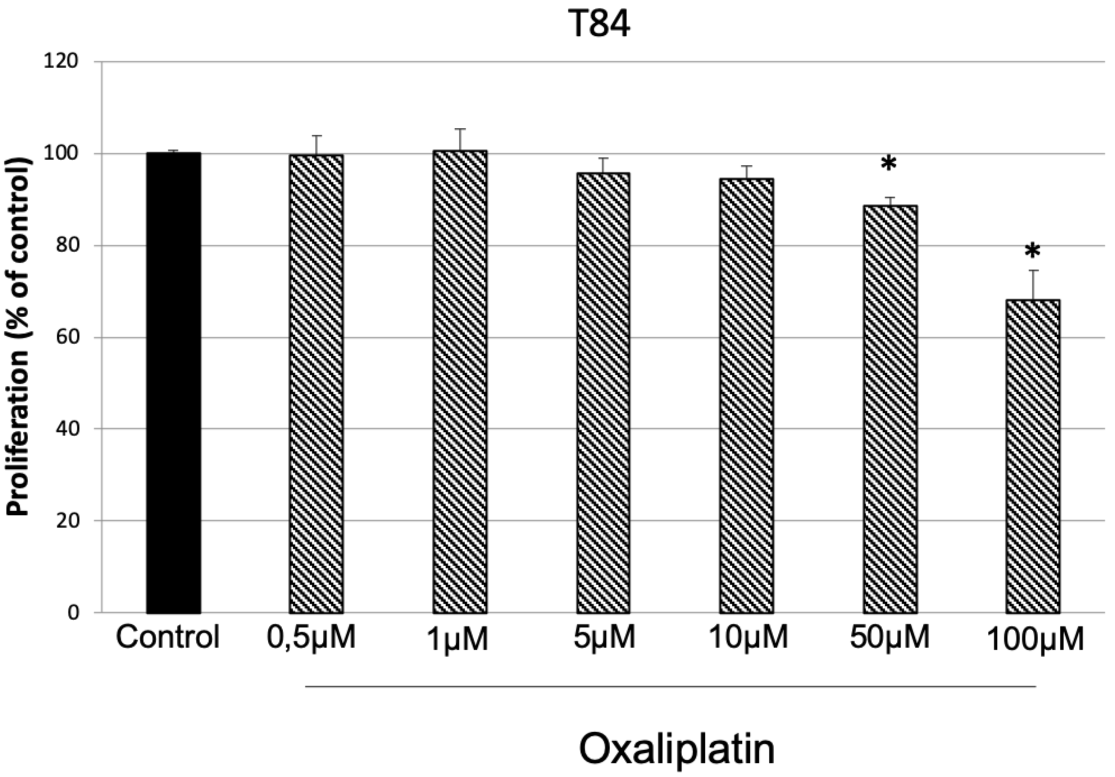

Figure 4. Influence of different Oxaliplatin concentrations on the proliferation of T84 cells. Oxaliplatin was added at the indicated concentrations for another 24 h. Mean values from at least three independent experiments are shown as mean ± SD. *$p < 0.05$.
